# The effects of brain endurance training on mental fatigue, endurance, and cognitive function: a systematic review and meta-analysis

**DOI:** 10.3389/fphys.2026.1746298

**Published:** 2026-06-03

**Authors:** Jialiang Yu, Zhiyuan Tan, Yuxiong Huang, Lunxin Chen, Danyang Li

**Affiliations:** 1 School of Sports Training, Wuhan Sports University, Wuhan, China; 2 Faculty of Sport and Physical Education, University of Belgrade, Belgrade, Serbia; 3 School of Physical Education and Sports, Central China Normal University, Wuhan, China

**Keywords:** attention, brain endurance training, decision making, endurance, inhibition, mental fatigue, meta-analysis

## Abstract

This study systematically evaluated the effects of Brain Endurance Training (BET) on alleviating mental fatigue, enhancing endurance performance, and improving cognitive function through a systematic review and meta-analysis of nine peer-reviewed articles comprising 11 studies identified via a comprehensive database search following PRISMA 2020 guidelines. The pooled results demonstrated that BET significantly improved endurance performance compared to control conditions, with a more pronounced effect under fatigued conditions (SMD = 0.87, 95% CI 0.61–1.13) than at baseline (SMD = 0.21, 95% CI 0.08–0.34). Cognitive performance measures also showed substantial improvements, including faster Stroop test reaction times (Stroop-RT) (MD = 56.62, 95% CI 16.50–96.73) and enhanced Psychomotor Vigilance Task reaction time (PVT-RT) both at baseline (MD = 1.96, 95% CI 0.15–3.77) and under fatigue (MD = 18.72, 95% CI 15.27–22.18). BET exhibited significantly greater mental fatigue resistance in fatigued states (MD = 9.22, 95% CI 1.64–16.79) compared to baseline (MD = -0.54, 95% CI -2.44–1.36). Subgroup analyses revealed that no statistically significant differences were observed between subgroups based on participant characteristics or training protocols (all p > 0.05). While some descriptive numerical trends were observed, suggesting that BET might be influenced by individual differences and training design, these findings should be interpreted with caution due to overlapping confidence intervals and limited statistical power. Specifically, point estimates appeared higher in untrained individuals (SMD = 0.32, 95% CI 0.12–0.52) than in trained individuals (SMD = 0.13, 95% CI −0.04–0.30). Similarly, BET yielded comparable improvements in dynamic endurance tests (SMD = 0.22, 95% CI 0.08–0.36) and static tests (SMD = 0.19, 95% CI −0.14–0.52). Numerically larger effects were also noted when cognitive training preceded physical training (SMD = 0.33, 95% CI 0.06–0.61). In addition, interventions lasting ≥ 8 weeks (SMD = 0.27) and lower-frequency training (< 4 sessions/week) showed descriptive tendencies toward greater improvements. Overall, these findings provide preliminary quantitative insights into the impact of BET and offer preliminary references for designing specialized training protocols.

**Systematic review registration:**

https://www.crd.york.ac.uk/prospero/, identifier CRD420251128193.

## Introduction

In modern society, technological advancements have led to a gradual shift toward cognitive labor in most occupations (Boksem and Tops, 2008). High-intensity cognitive tasks are becoming increasingly prevalent, and mental fatigue has become a common health issue (Boksem and Tops, 2008; Grillon et al., 2015; Daneshgar-Pironneau et al., 2025). Mental fatigue typically occurs after prolonged, high-intensity training or tasks requiring sustained attention (André et al., 2025; Oliver et al., 2025; Weiler et al., 2025), manifesting as a decline in energy levels, slower thinking speed, and reduced attention (Boksem and Tops, 2008; Marcora et al., 2009; Li et al., 2020), among other cognitive impairments (Yang et al., 2013; Hopstaken et al., 2015; Van Cutsem et al., 2017; Liu et al., 2025). This condition not only affects various aspects of daily life (Van Cutsem et al., 2017; Zhang et al., 2021), such as impairing balance control in the elderly and increasing the risk of falls (Nikooharf Salehi et al., 2023; Pitts and Bhatt, 2023; Zhang et al., 2023; Díaz-García et al., 2025), but also significantly diminishes endurance performance, negatively impacting endurance (Van Cutsem et al., 2017; Flemington et al., 2024) and dynamic motor execution (Smith et al., 2016; Thompson et al., 2019; Mariano et al., 2023).

Mental fatigue has become increasingly prevalent (Ricci et al., 2007). For example, in the Netherlands, nearly half of women and one-third of men report experiencing mental fatigue, a significant increase compared to 15 years ago. In the United States, 38% of workers report fatigue, with 66% noting a decrease in their work productivity (Ricci et al., 2007). Furthermore, mental fatigue is a common symptom of various chronic diseases (Boksem and Tops, 2008), including HIV/AIDS (Campbell et al., 2022), multiple sclerosis (Boksem and Tops, 2008), and neurodegenerative diseases (Chaudhuri and Behan, 2000). Thus, developing effective interventions and optimizing their implementation is urgently needed.

Brain Endurance Training (BET), as an emerging intervention, was introduced by Marcora et al. (2015) in 2015. This method involves dual cognitive and physical loads designed to build mental fatigue resilience, improve performance, and enhance performance-related fatigue resistance. Unlike exercise-cognitive combined training (ECCT), which primarily targets the improvement of quality of life (Pellegrini-Laplagne et al., 2023), and fall prevention in older adults (Khan et al., 2025). BET specifically focuses on enhancing psychophysical performance by increasing an individual’s tolerance to mental fatigue (André et al., 2025). Studies have shown that BET not only significantly improves endurance performance (the experimental group showed a 113% improvement in performance during exhaustive tests, compared to 43% in the control group), but also enhances cognitive functions related to working memory. For example, Dallaway et al (Dallaway et al., 2021; Dallaway et al., 2024). found that BET improved cognitive reaction speed in dynamic gymnastics athletes. Similarly, Díaz-García et al (Díaz-García et al., 2023). reported that BET improved decision speed and accuracy in paddle sports athletes, with these improvements closely linked to enhanced visual attention and decision-making abilities (Lucia et al., 2021). Additionally, Staiano et al. (2025) demonstrated that BET could maintain cognitive performance even under fatigue, suggesting its protective role in complex cognitive tasks such as tactical decision-making and attention allocation during sports activities.

Although existing experimental studies support the effectiveness of BET, the results remain inconsistent, possibly due to differences in study design, experimental conditions, and participant characteristics. For example, the impact of training levels on the effectiveness of BET is still unclear (Díaz-García et al., 2023; Dallaway et al., 2023; Díaz-García et al., 2024), and the sequencing of cognitive tasks within the training plan (e.g., pre-training, simultaneous, post-training, or alternating) may also influence the outcomes (André et al., 2025). Therefore, while the value of BET is acknowledged, further exploration is necessary to understand the underlying mechanisms for the differences in outcomes, in order to provide more targeted recommendations for future applications.

To date, André et al. (2025) were the only researchers who have conducted a systematic review of the effects of BET. The study concluded that BET is a promising intervention that may reduce mental fatigue and improve endurance and cognitive performance. However, they also pointed out that the effects of BET on subjective mental fatigue are still inconclusive. Although the study examined factors such as training frequency, duration, type, and the integration of cognitive and physical training, the impact of these variables has not been quantitatively analyzed.

Compared to individual studies, meta-analysis enhances statistical power, providing more precise and robust conclusions (Jackson and Turner, 2017). However, without subgroup or meta-regression analysis, we cannot deeply understand how participant characteristics and intervention specifics might modulate the effects of BET. Given that factors such as gender, training status, and training protocols may significantly affect the adaptation to BET, it is crucial to explore how these variables influence the outcomes. This understanding will help practitioners design BET programs tailored to individual differences, ensuring their applicability and effectiveness. To date, no meta-analysis has systematically examined the effects of BET (Trafford, n.d.), nor has there been a thorough investigation into how participant characteristics and training parameters modulate these effects.

Therefore, this study aimed to systematically evaluate the effects of BET on endurance, mental fatigue, and cognitive functions (e.g., executive function measured by the Psychomotor Vigilance Task reaction time, PVT-RT) under both fresh and fatigued conditions. Additionally, we will explore potential dose-response relationships and examine how participant characteristics and training parameters influence these outcomes, providing empirical evidence for the development of personalized BET protocols.

## Methods

### Design

This systematic review follows the Preferred Reporting Items for Systematic Reviews and Meta-Analyses (PRISMA) statement (Higgins et al., 2024). Additionally, this review has been registered in PROSPERO and was submitted on August 19, 2025 (ID: CRD420251128193).

### Eligibility criteria

According to the PICOS criteria for study inclusion (**Table 1**), only studies involving healthy participants were included. The primary reason for selecting a healthy population is to eliminate potential interference from pre-existing health conditions or diseases that could affect the outcomes of BET. Healthy individuals provide a more stable baseline, allowing for a more accurate assessment of the effects of BET on physical performance, cognitive function, and mental health. Additionally, no age restrictions are imposed in this study, aiming for a more comprehensive evaluation of the effects of BET across different age groups, and exploring its potential benefits at various stages of the lifespan for physical performance, cognitive function, and mental endurance. Furthermore, dual-task studies were excluded because their optimization objectives differ from BET, which specifically aims to induce mental fatigue to foster adaptation (Pellegrini-Laplagne et al., 2023; André et al., 2025; Khan et al., 2025).

**Table 1:** PICOS eligibility inclusion and exclusion criteria.

Category	Inclusion criteria	Exclusion criteria
Population	Healthy individuals, with no restrictions on age, sex, or training status	Disease populations
Intervention	Implementation of brain endurance training interventions aimed at improving resistance to MF and improve psychophysical performance.	Single-mode training (cognitive or physical only) or non-fatigue-inducing dual-task interventions
Comparative intervention	Control group is either a no-intervention group, a cognitive intervention group, or a physical training group	Studies without a control group were excluded
Outcome	At least one measure related to endurance, mental fatigue-related indicators or cognitive function-related indicators like inhibitory control and attention	Lack of baseline data
Study design	Randomized controlled trials (RCTs), including parallel-group and crossover designs	Single-group interventions or non-randomized controlled trials

### Information sources

Before the formal search, one of the authors (JLY) performed a preliminary search of related articles on Brain Endurance Training (BET) in the Web of Science database and developed the search terms. The search terms were designed to include as many manually retrieved papers as possible. On July 27, 2025, a comprehensive search was conducted in Web of Science (WOS), PubMed, and Scopus for peer-reviewed English literature related to Brain Endurance Training.

### Search strategy

A comprehensive systematic search was conducted across electronic databases, including Web of Science, PubMed, and Scopus. For example, the search string for Web of Science was as follows: (((((((((TS=(“Endurance Performance”)) OR TS=(“Physical Endurance”)) OR TS=(“Exercise Performance”)) OR TS=(“Athletic Performance”)) OR TS=(“Performance”)) OR TS=(“Sport Performance”)) OR TS=(“Aerobic Capacity”)) OR TS=(“Physical Capacity”)) OR TS=(“Mental Fatigue”)) OR TS=(“Cognitive Fatigue”) AND ((((TS=(“Brain training”)) OR TS=(“BET”)) OR TS=(“brain endurance training”)) OR TS=(“Cognitive Training”)) OR TS=(“Mental Endurance”). During the search in the Web of Science database, the “Document Type” filter was applied to initially exclude conferences, reviews, abstracts, patents, editorials, letters, corrections, news, theses, unreviewed articles, case reports, and other non-relevant document types. Detailed search strategies tailored for each specific database are provided in the Electronic **Supplementary Material (Appendix S2)**.

### Selection process

Two authors (JLY and ZYT) independently screened the titles, abstracts and full-text of these selected studies. Reference lists were also examined from the selected articles to be included to identify any additional relevant studies. The screening process removed articles at each stage according to the inclusion and exclusion criteria, including duplicates, assessing the title, screening the abstract, and reviewing the full text. A third author (YXH) adjudicated non-resolvable disagreements.

### Data extraction

The database search results were imported into the bibliographic management software (Endnote X9, Clarivate INC, Philadelphia, PA 19130, USA), and duplicate copies were removed. If data were missing or presented only in graphical form, the authors were contacted to obtain the necessary information. Getdata was used to extract the relevant data (Zein et al., 2015). Studies with missing data that could not be obtained were excluded from the final analysis. The extracted data included pre- and post-intervention mean values, standard deviations, and sample sizes.

A standardized data extraction worksheet was created in Excel prior to the full-text screening process. For studies with missing data or those providing only graphical results, the original authors were contacted to request additional information. If data could not be obtained through correspondence author, Getdata was used to extract values from graphical figures (Zein et al., 2015). Studies with unresolved missing data were excluded from the final analysis.

The extracted information included the following key elements: authors’ names and the year of publication; participant characteristics such as sport type, age, sample size, and training level; details of the interventions, including the type of training, timing of cognitive task assignments, frequency, and duration; and outcome measures, which comprised the mean values, standard deviations, and standard errors of each indicator. Data were collected for both the intervention and control groups under two conditions: fresh vs. fatigued. Endurance performance indicators included time trial results, exhaustion time, and the number of exhaustion events. Cognitive outcomes were measured based on reaction time, and subjective indicators related to mental fatigue were also extracted.

### Statistics and data synthesis

Statistical analysis and plotting were performed using the “meta” and “metafor” packages in software R (version 4.3.3). To evaluate the effectiveness of Brain Endurance Training (BET) in combating fatigue, some studies employed analysis of variance (ANOVA). Specifically, some studies (Staiano et al., 2022; de Lima-Junior et al., 2023; Staiano et al., 2023; Dallaway et al., 2024) examined the group (BET vs. CON) × time (pre-intervention vs. post-intervention) interaction, focusing on the changes within each group before and after the intervention. Other studies (Díaz-García et al., 2023; Dallaway et al., 2023; Díaz-García et al., 2024; Díaz-García et al., 2025; Staiano et al., 2025) expanded the analysis by incorporating the group (BET vs. CON) × time (pre-intervention vs. post-intervention) × condition (fresh vs. fatigue intervention) interaction, which allowed for the comparison of endurance and cognitive task performance across different conditions. It is important to note that some studies only performed the group × time interaction analysis, which compared pre- and post-intervention changes, without considering the additional impact of fatigue interventions on endurance and cognitive performance.

From each study, we extracted data on endurance and cognitive task performance for both the experimental and control groups under different states (fresh vs. fatigued). As some studies reported results separately for fresh and fatigued states, we performed independent analyses for each state to ensure the accuracy of the comparisons.

We then extracted the mean change (Mchange) and standard deviation change (SDchange), calculated based on the following formulas:


Mchange=Mpost−Mpre

$$\text{SDchange}=\sqrt{{\text{SD}}_{\text{pre}}^{2}+{\text{SD}}_{\text{post}}^{2}-2\times \text{r}\times {\text{SD}}_{\text{pre}}\times {\text{SD}}_{\text{post}}}$$

Mpre and Mpost represent the means before and after the intervention under different conditions (fresh vs. fatigue intervention), while SDpre and SDpost correspond to the standard deviations for each condition. When studies reported the standard error (SE), it was converted to the standard deviation (SD), the following formula is used for conversion:


$$\begin{array}{l} \text{SD }=\text{ SE}\times \sqrt{\text{n}} \end{array}$$

r is the correlation coefficient. In cases where the correlation coefficient (Corr) was not explicitly reported in the studies, it was either calculated through correlation analysis based on raw data or obtained through communication with the original research teams. If these methods were not feasible, Corr was assumed to be 0.5 based on the Cochrane Handbook (Higgins et al., 2024).

For endurance-related measures, due to differences in evaluation units and test distances between studies, we used the Standardized Mean Difference (SMD) and its 95% Confidence Interval (CI) as the effect size for the results. The magnitude of the SMD was interpreted using Cohen’s criteria: < 0.2 (trivial), 0.2–0.49 (small), 0.5–0.8 (medium), and > 0.8 (large) (Cohen, 2009). For reaction times and mental fatigue-related measures, since the evaluation units were the same across studies, we used the Mean Difference (MD) and its 95% CI as the effect size for these results. Prediction intervals (PI) were calculated to more comprehensively reflect the potential variability of similar studies in the future (Nagashima et al., 2019).

The Cochrane Handbook does not provide a unified recommendation for model selection. Considering the differences in the design of BET training protocols, interventions, and populations, to avoid the potential for the fixed-effect model to overlook heterogeneity, we used the random-effects model recommended by DerSimonian and Laird (DerSimonian and Kacker, 2007). This model assumes that the effect sizes come from a distribution of true effects rather than a single homogeneous group. By assuming that the underlying effects follow a normal distribution, the random-effects model can incorporate heterogeneity, thus providing a more accurate estimate of the overall effect size.

The model parameters were estimated using the Restricted Maximum Likelihood (REML) method and cross-validated against the Maximum Likelihood (ML) method to ensure result stability. For all models, individual coefficients and their corresponding confidence intervals (CIs) were tested based on the t-distribution (Jukic et al., 2023). For the assessment of heterogeneity, commonly used statistics include the Cochrane Q test, I² statistic, and tau² (Nakagawa et al., 2017). However, most literature tends to use I² as the main heterogeneity assessment metric. Therefore, in the primary analysis, we reported I² and interpreted it based on the following criteria: < 25% indicates low heterogeneity, 25%–75% indicates moderate heterogeneity, and > 75% indicates high heterogeneity (Higgins and Thompson, 2002). When the p-value is less than 0.05, it indicates significant heterogeneity. To minimize the risk of Type I error inflation, we established a hierarchical structure for all outcome measures (Bender and Lange, 2001). Primary outcomes for confirmatory analysis were predefined as the non-fatigued state assessments of subjective mental fatigue, endurance capacity, and cognitive performance (Stroop and PVT-RT). Conversely, all tests performed in the fatigued state, along with subgroup analyses and meta-regressions, were categorized as secondary or exploratory outcomes.

### Subgroup and meta-regression analysis

This study employed both subgroup and meta-regression analyses to statistically examine categorical and continuous variables. The subgroup analysis was conducted based on several factors, including training status (whether participants had more than two years of training experience), outcome measures (dynamic vs. static), cognitive task scheduling (Pre-BET, Post-BET, Current-BET, and Mixed-BET), gender, and key training parameters: training duration (< 8 weeks vs. ≥ 8 weeks) and training frequency (< 4 sessions/week vs. ≥ 4 sessions/week). These analyses aimed to assess the impact of different variables on the outcomes.

We also performed mixed-effects meta-regression analysis using restricted maximum likelihood estimation (REML), which is recognized for its robustness (Christ, 2010). The screening variables included the BET training protocol: (a) total duration of each training session; (b) frequency of training per week; (c) total training duration per week; (d) number of training weeks; (e) total training duration; (f) duration of each physical training session; (g) duration of each cognitive training session; (h) total number of training sessions; (i) age. To assess the form of the relationship, we fitted both linear and nonlinear functions, compared these models, and selected the one with the lowest Akaike Information Criterion (AIC) correction for bias (Harrell, 2015). All regression models were conducted using the metafor package and subsequently visualized using the ggplot2 package (Wickham, 2011).

### Risk of bias assessment

Two researchers (JLY and ZYT) independently assessed the risk of bias in the included studies and cross-verified the results. The risk of bias was evaluated using the RCT risk of bias assessment tool recommended by the Cochrane Handbook 5.1.0 (Higgins et al., 2024), and the quality of the studies was assessed using Review Manager 5.4.

The assessment covered random sequence generation, random allocation concealment, blinding of outcome assessment, completeness of outcome data, and selective reporting of outcomes. Additionally, the Physiotherapy Evidence Database (PEDro) scale (De Morton, 2009) was used to evaluate the risk of bias and methodological quality of the included studies. The PEDro scale evaluates studies on a scale from 0 to 10, with scores of 6 and above considered high quality, 4–5 as moderate quality, and 3 or below as low quality.

### Publication bias analysis and sensitivity analyses

To evaluate publication bias, we employed a contour-enhanced funnel plot (Peters et al., 2008), with a *p*-value greater than 0.05 indicating no significant risk of publication bias. Both the funnel plot and Egger’s regression test were primarily used to assess the symmetry of the overall effect size, providing both a visual and statistical evaluation of the potential for publication bias in the included studies.

Additionally, a sensitivity analysis was conducted by sequentially excluding individual studies to determine whether any single study exerted a disproportionate influence on the overall pooled effect size.

## Results

### Study selection and study characteristics

**Figure 1:**
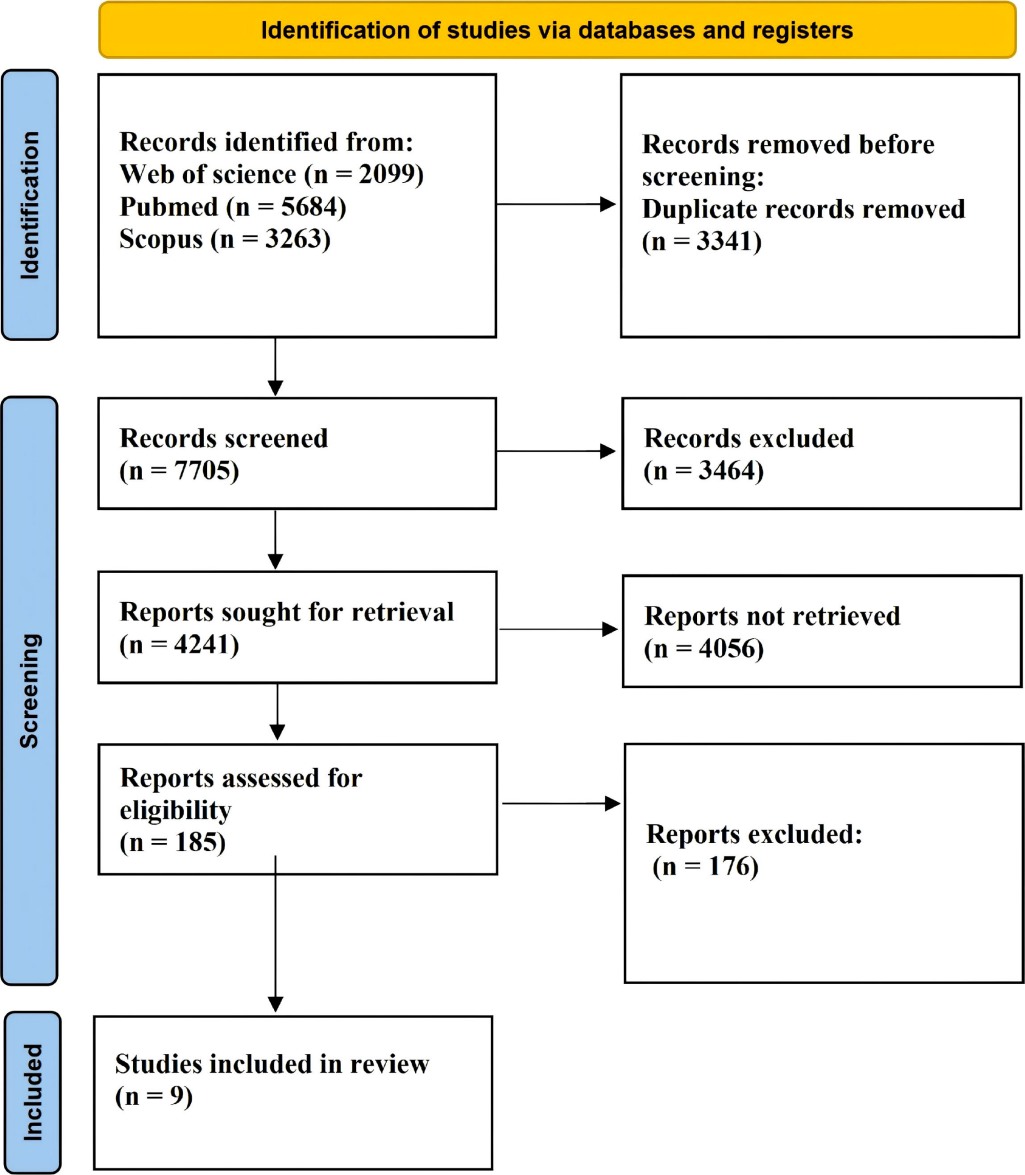
PRISMA flow diagram detailing the study inclusion process.

A total of 11,046 studies were initially identified, with 3,263 from Scopus, 5,684 from PubMed, and 2,099 from the Web of Science databases. After applying filters based on article type and experimental design, 7,582 studies remained. Following the removal of duplicates using Zotero’s duplicate detection function, and additional manual elimination, 4,241 studies were retained. A review of titles and abstracts led to the identification of 185 potentially relevant articles. Subsequent full-text screening resulted in the exclusion of 176 studies, leaving 9 articles (Staiano et al., 2022; Díaz-García et al., 2023; Dallaway et al., 2023; de Lima-Junior et al., 2023; Staiano et al., 2023; Díaz-García et al., 2024; Dallaway et al., 2024; Díaz-García et al., 2025; Staiano et al., 2025), encompassing a total of 11 studies, that met the inclusion criteria. The article inclusion process is summarized in **Figure 1**.

Notably, in the study by Dallaway et al. (2024), data from two separate studies were extracted. The 11 included studies reported a combined total sample size of 438 participants, with 177 in the experimental group and 261 in the control group. All participants were healthy adults, aged between 14 and 78 years. The interventions varied across studies: two studies administered cognitive training before physical training (Pre-BET), two studies conducted cognitive training after physical training (Post-BET), one study combined cognitive and physical training simultaneously (Current-BET), and five studies integrated cognitive training within the physical training regimen (Mix-BET).

In these studies, the experimental group received Brain Endurance Training (BET), while the control group engaged in alternative training modalities. In one study, the control group participated in tactical and technical training, while the other 10 studies used physical training as the control condition. Notably, in the study by de Lima-Junior et al (de Lima-Junior et al., 2023), the control group consisted of two subgroups: one performing cognitive training and the other undergoing physical training. The duration of interventions across all studies ranged from 4 to 12 weeks, with training sessions occurring 2 to 5 times per week. Detailed bibliographic information is provided in **Appendix S1**.

### Methodological appraisal of the included studies

**Figure 2:**
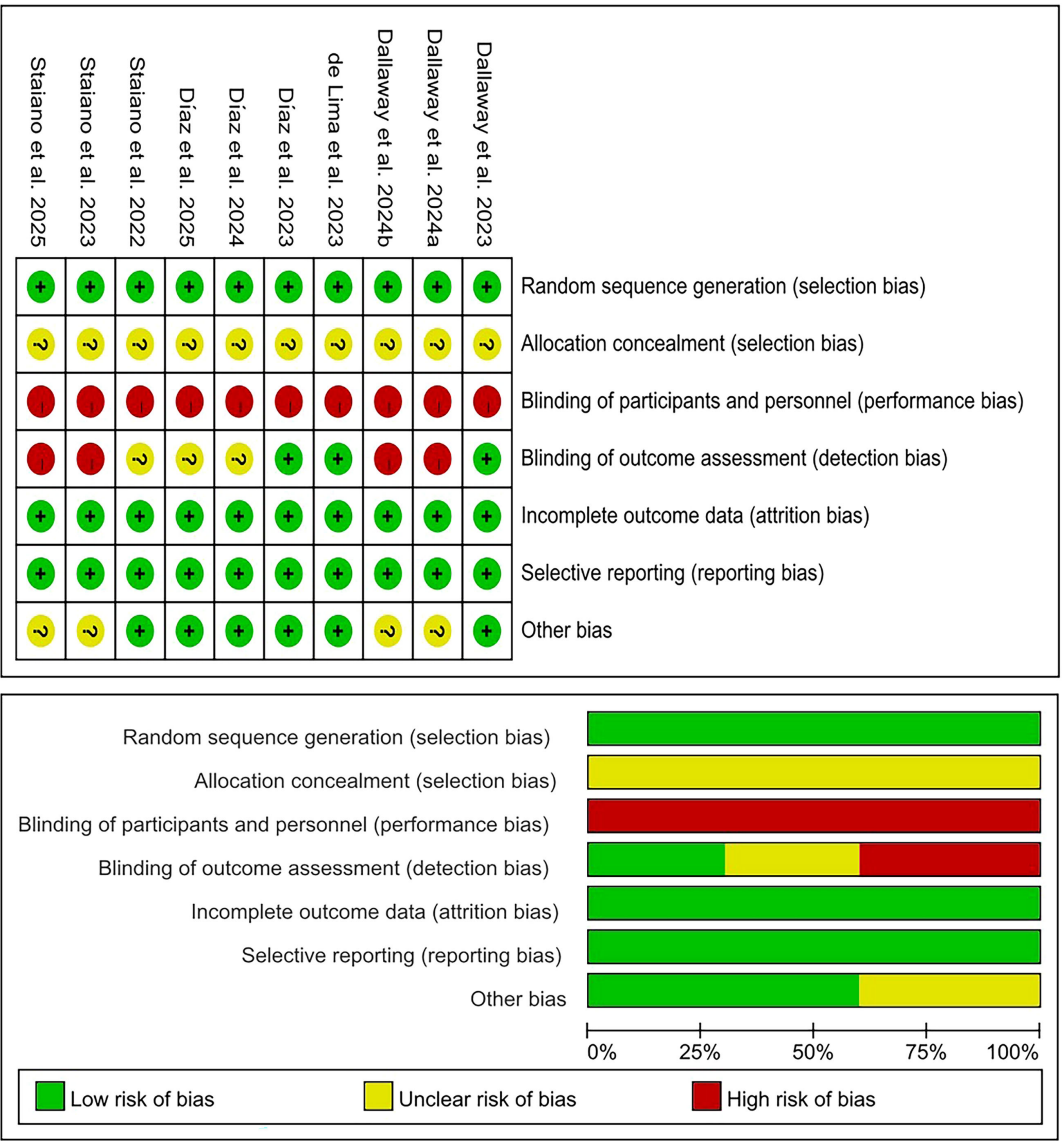
Risk of bias assessment for all included studies.

The results of the risk of bias assessment for the included studies are presented in **Figure 2**. Due to limitations in experimental design, none of the studies employed a double-blind design, resulting in all studies being rated as “high risk” for this domain. However, all studies utilized randomization methods, which were rated as “low risk.” The issue of allocation concealment was unclear in all studies, leading to an “unclear risk” rating for this criterion.

Regarding “incomplete outcome data” (attrition bias) and “selective reporting” (reporting bias), all studies were classified as “low risk.” In terms of “blinding of outcome assessment” (detection bias), three studies (Díaz-García et al., 2023; Dallaway et al., 2023; de Lima-Junior et al., 2023) were rated as “low risk,” four studies (Staiano et al., 2022; Díaz-García et al., 2024; Díaz-García et al., 2025) were rated as “unclear risk,” and three articles involving four studies (Staiano et al., 2023; Dallaway et al., 2024; Staiano et al., 2025) were rated as “high risk.” For “other bias,” three articles involving four studies (Staiano et al., 2023; Dallaway et al., 2024; Staiano et al., 2025) were rated as “unclear risk,” while the remaining six studies (Staiano et al., 2022; Díaz-García et al., 2023; Dallaway et al., 2023; de Lima-Junior et al., 2023; Díaz-García et al., 2024; Díaz-García et al., 2025) were rated as “low risk.” Overall, the methodological quality of the included studies was low. While randomization and data reporting were rigorous (low risk), the universal lack of participant and personnel blinding represents a significant source of performance bias. Although the average PEDro score was 8.3 (**Appendix S3**), this does not fully mitigate the risk that observed effects could be influenced by participant expectations.

### Risk of bias assessment

**Figure 3:**
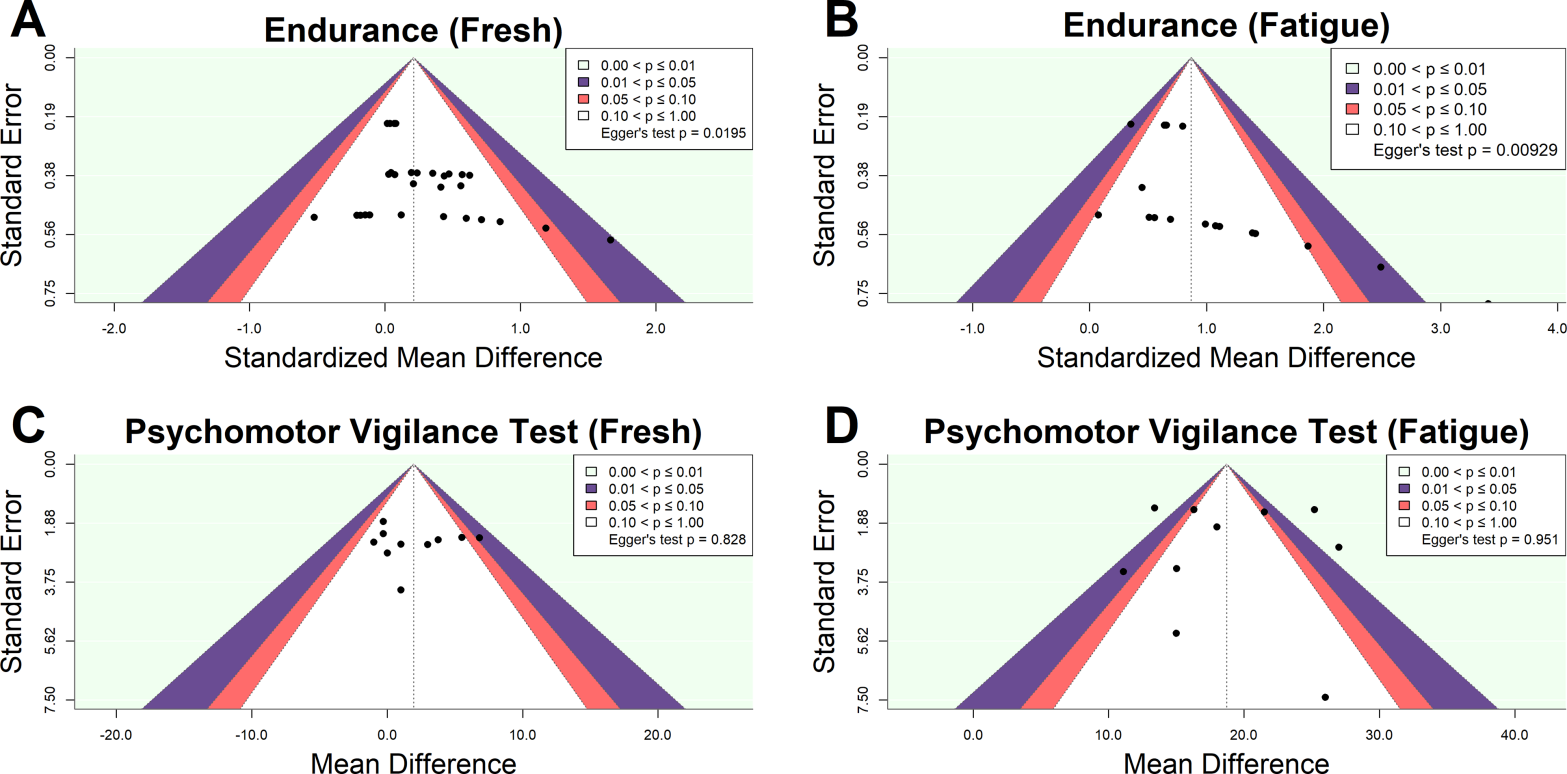
Visualization of publication and bias, Egger’s test. **(A)** Endurance in a fresh state; **(B)** Endurance in a fatigued state; **(C)** PVT-RT in a fresh state; **(D)** PVT-RT in a fatigued state).

Funnel plot combined with Egger’s test was used to assess publication bias (**Figure 3**). Due to the limited number of studies, the analysis focused on the impact of the included studies on endurance and PVT-RT outcomes in both the fresh and fatigued states. The results of the Egger’s test indicated a potential risk of publication bias for endurance tests in both the fresh (*p* = 0.02) and fatigued (*p* < 0.05) states. However, no publication bias was detected for the PVT-RT test in either the fresh or fatigued states.

### Sensitivity analysis

A sensitivity analysis was conducted by sequentially removing each study to assess the influence of individual studies on the overall combined effect. Our findings revealed that excluding certain studies resulted in significant changes in the summary results. For the mental fatigue test in the fatigued state, removing studies by Staiano et al. (2025) (k = 5, ES = 7.70, *p* = 0.0710) and Díaz et al (Díaz-García et al., 2024). (k = 5, ES = 9.12, *p* = 0.06) caused the summary result to shift from significant to non-significant. Similarly, for the PVT-RT, excluding Díaz et al (Díaz-García et al., 2024). with training durations of four weeks (k = 9, ES = 1.5203, *p* = 0.1) and eight weeks (k = 9, ES = 1.3317, *p* = 0.1), as well as Díaz et al (Díaz-García et al., 2023). with training durations of three weeks (k = 9, ES = 1.8579, *p* = 0.07) and eight weeks (k = 9, ES = 1.7534, *p* = 0.08), resulted in the summary analysis shifting from significant to non-significant. For the Stroop test, removing Staiano et al. (2022) (k = 3, ES = 55.05, *p* = 0.09) caused the summary result to transition from significant to non-significant.

### Meta-analysis

**Figure 4:**
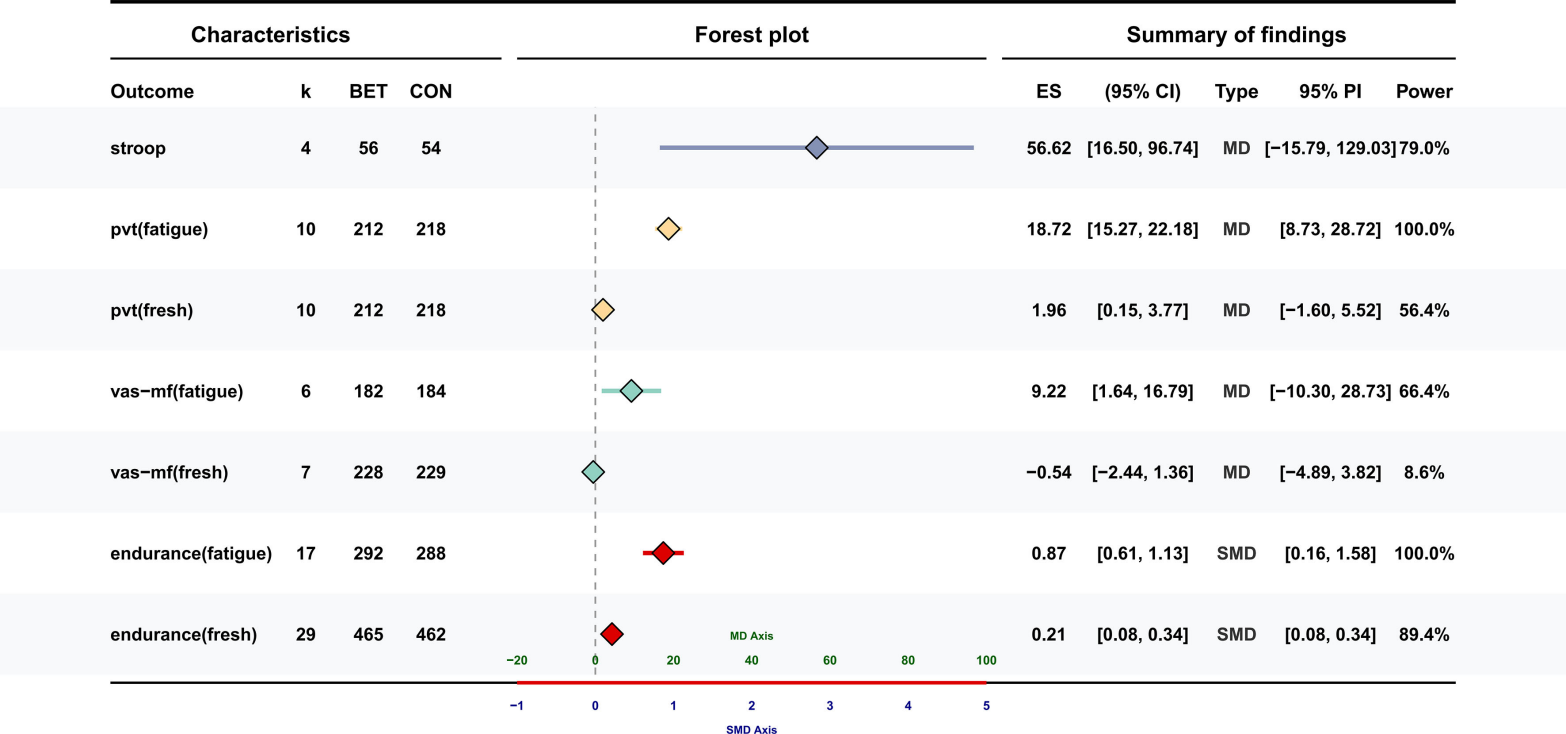
Combined forest plot of the effects of brain endurance training (BET) on various outcomes. The plot displays pooled effect sizes (ES) across cognitive (Stroop, PVT, VAS-MF) and physical (Endurance) outcomes. Characteristics: k denotes the number of studies; BET/CON represents sample sizes. Forest Plot: Diamonds and error bars represent pooled ES and 95% CIs, respectively. The green axis indicates Mean Difference (MD), while the blue axis indicates Standardized Mean Difference (SMD). Summary of Findings: 95% PI (Prediction Interval) represents the expected range of effects in future settings, accounting for heterogeneity. ‘Fatigue’ and ‘fresh’ denote outcomes measured under induced mental fatigue or rested states, respectively.

A total of 6 studies (Dallaway et al., 2023; de Lima-Junior et al., 2023; Staiano et al., 2023; Díaz-García et al., 2024; Dallaway et al., 2024; Díaz-García et al., 2025) were included, yielding 29 effect sizes that assessed the impact of BET on endurance capacity (**Figure 4**). The meta-analysis revealed that, compared to the control group, BET significantly improved endurance levels in the fresh state (SMD = 0.21, 95% CI 0.08–0.34), with a small effect size. No heterogeneity was observed across studies (I² = 0%, *p* > 0.05). In the fatigued state, 3 studies (Staiano et al., 2023; Díaz-García et al., 2024; Díaz-García et al., 2025) and 17 effect sizes evaluated the impact of BET on endurance (**Figure 4**). The meta-analysis demonstrated that, compared to the control group, BET resulted in a large effect size (SMD = 0.87, 95% CI 0.61–1.13) in improving endurance levels in the fatigued state, with moderate heterogeneity observed between studies (I² = 45.1%, *p* < 0.05).

For mental fatigue, 3 studies (Díaz-García et al., 2023; Díaz-García et al., 2024; Staiano et al., 2025) with 7 effect sizes evaluated the impact of BET on mental fatigue in the fresh state (**Figure 4**). After taking the negative value of the effect size, the meta-analysis showed that there was no significant difference in mental fatigue between the BET group and the control group (MD=-0.54, 95% CI -2.44–1.36), with moderate heterogeneity (I²=62%, *p* < 0.05). For mental fatigue in the fatigued state, 3 studies (Díaz-García et al., 2023; Díaz-García et al., 2024; Staiano et al., 2025) with 6 effect sizes evaluated the impact of BET (**Figure 4**). The meta-analysis showed that BET significantly improved mental fatigue in the fatigued state (MD = 9.215, 95% CI 1.637–16.794), with large heterogeneity (I²=96.4%, *p* < 0.001).

For Stroop reaction time (Stroop-RT), 3 studies (Staiano et al., 2022; de Lima-Junior et al., 2023; Staiano et al., 2023) with 4 effect sizes evaluated the impact of BET on Stroop-RT in the fresh state (**Figure 4**). After taking the negative value of the effect size, the meta-analysis showed that BET significantly improved Stroop-RT performance in the fresh state (MD = 56.619, 95% CI 16.50–96.73), with moderate heterogeneity (I²=66.9%, *p* < 0.05).

For PVT-RT, 4 studies (Díaz-García et al., 2023; Díaz-García et al., 2024; Díaz-García et al., 2025; Staiano et al., 2025) with 10 effect sizes evaluated the impact of BET on PVT-RT in the fresh state (**Figure 4**). After taking the negative value of the effect size, the meta-analysis showed that BET significantly improved PVT-RT in the fresh state (MD = 1.959, 95% CI 0.15–3.77), with trivial heterogeneity (I²=29.1%, *p* > 0.05). For the fatigued state, 4 studies (Díaz-García et al., 2023; Díaz-García et al., 2024; Díaz-García et al., 2025; Staiano et al., 2025) with 10 effect sizes evaluated the impact of BET on PVT-RT (**Figure 4**). The meta-analysis showed that BET significantly improved PVT-RT in the fatigued state (MD = 18.72, 95% CI 15.27–22.18), with large heterogeneity (I²=84.3%, *p* < 0.001).

### Subgroup analysis

**Figure 5:**
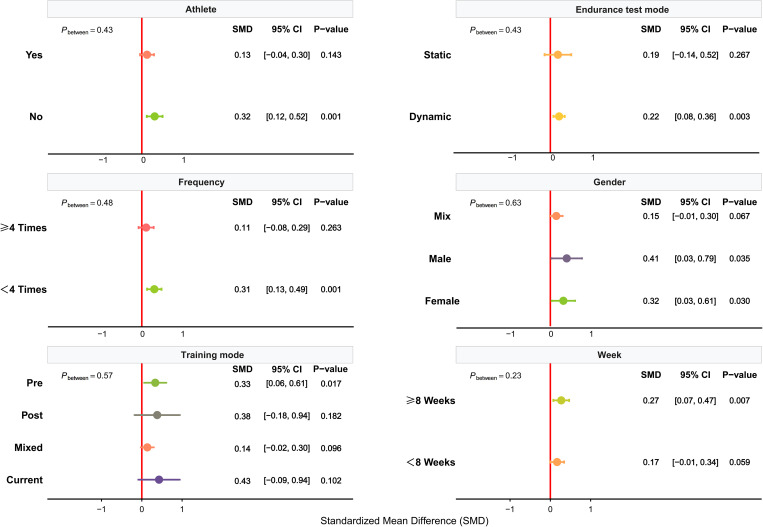
Subgroup analysis of brain endurance training (BET) effects on endurance performance in a fresh state. Effect size (points) reflects the Standardized Mean Difference (SMD) of the BET effect, with horizontal bars representing the 95% Confidence Interval (CI). The vertical red line indicates the null effect (SMD = 0). *P*_between values displayed at the top left of each panel indicate the significance of the moderating effect between subgroups. Categorization details: Athlete: “Yes” refers to competitive athletes; “No” refers to recreationally active or untrained individuals. Endurance test mode: “Dynamic” refers to whole-body exercise (e.g., cycling, running); “Static” refers to isometric tasks (e.g., sustained contractions). Frequency: Subgroups divided by weekly sessions: “≥4 Times” and “< 4 Times”. Gender: “mix” (mixed gender samples), “m” (male), and “f” (female). Training mode: Order of cognitive and physical tasks. “Pre” (cognitive before physical), “Post” (cognitive after physical), “Mixed” (varied order), and “Current” (simultaneous cognitive and physical tasks). Week: Duration of intervention: “≥ 8 Weeks” and “< 8 Weeks”.

No statistically significant differences were observed in any of the subgroups (*p* > 0.05) (**Figure 5**). However, in specific circumstances, BET showed significant advantages in improving endurance in the fresh state compared with the control group. Gender Subgroup: Subgroup analysis revealed that BET had a positive effect on both males (ES = 0.41) and females (ES = 0.32). However, the test for subgroup differences indicated that there was no statistically significant difference between genders (*p* = 0.63). Furthermore, the effect size for the mixed-gender group was notably lower (ES = 0.15) and did not reach statistical significance. Training Status: BET only showed a significant advantage in untrained individuals (ES = 0.32). Test Type: Only dynamic testing indicators (ES = 0.32) showed significant advantages. Cognitive Training Timing: Only the pre-BET (ES = 0.33) group showed a significant advantage. Among training-related moderators, interventions lasting ≥ 8 weeks led to greater improvements in endurance performance (SMD = 0.27), whereas shorter interventions (< 8 weeks) had a smaller effect on endurance (SMD = 0.17). Lower-frequency training regimens (< 4 sessions/week) were associated with a significant improvement in endurance performance (SMD = 0.31). In contrast, higher-frequency training (≥ 4 sessions/week) resulted in a smaller, non-significant effect (SMD = 0.11).

### Meta-regression analysis

Meta-regression analysis was also performed to examine the moderating effects of age, total training weeks, weekly training frequency, duration of physical training per session, duration of cognitive training per session, total session duration, total weekly training duration, total training duration, and total training sessions. However, no significant relationships were found between any of the training variables and the effects of BET on endurance in the fresh state (*p* > 0.05 for all). The specific information can be found in **Appendix S4**.

## Discussion

To the best of our knowledge, this study is the first systematic review and meta-analysis aimed at investigating the effects of Brain Endurance Training (BET) on endurance performance, cognitive function, and subjective fatigue in individuals under different conditions (i.e., fresh vs. fatigued). To enhance conceptual clarity and better interpret the efficacy of Brain Endurance Training (BET), we categorized the research outcomes into three distinct dimensions: (1) Subjective fatigue, assessed via self-report scales; (2) Endurance and cognitive performance, representing baseline functional capacity; and (3) Performance fatigability, referring to the decline in performance specifically induced by cognitive or physical load. We also performed a subgroup analysis on endurance performance to provide a comprehensive reference for related researchers and practitioners.

Our findings suggest that BET could be a potential strategy for combating mental fatigue and enhancing both endurance performance and cognitive abilities. Specifically, the BET group showed significant improvements in endurance performance, Stroop test response time, and PVT-RT. These cognitive gains exhibit distinct functional specificity and state-dependency: in terms of executive control, the BET group demonstrated a significant advantage in the Stroop task with an average reduction of 56.62 ms in response time. According to the meta-analysis by Mann et al. (2007) (Mann et al., 2007), the core competitive advantage of expert-level athletes in complex decision-making tasks involving inhibitory control often manifests within such tens-of-milliseconds differences. Regarding basic cognitive function, PVT results further corroborated this trend; the BET group showed a robust gain of 18.72 ms under fatigued conditions, while only a minimal change of 1.96 ms was observed in the fresh state. This “fatigue-dependent” pattern supports the positioning of BET as a “fatigue resilience” training tool, suggesting its value lies in maintaining functional stability when cognitive resources are near exhaustion rather than merely increasing baseline reaction limits. However, a cautious interpretation is warranted when considering the 95% prediction intervals (PI). While the PI for endurance performance remained entirely above zero, suggesting high replicability, the wider PI for the Stroop task which crosses zero suggests significant inter-individual heterogeneity. Therefore, while the average effects are significant and practically meaningful, future applications should account for differences in baseline cognitive capacity or task load, necessitating customized training protocols to optimize intervention outcomes. In the fatigued condition, the mental fatigue test also revealed a marked advantage for the BET group, while no substantial improvements were observed in subjective fatigue tests in the fresh state. Notably, the experimental group exhibited larger effect sizes in tests conducted under fatigued conditions compared to those in the fresh state, including endurance, PVT-RT, and mental fatigue improvement. This aligns with the hypothesis proposed by Andre et al (André et al., 2025), which suggests that the control group exhibits a greater decline in performance—from baseline to post-intervention—when tested under mental fatigue. In other words, while the control group’s performance is significantly impaired by cognitive load (showing a large negative disparity), the BET group demonstrates greater performance stability and resistance to fatigue-induced decrements. This disparity may be attributed to the impact of mental fatigue on performance assessments (Boksem and Tops, 2008; Marcora et al., 2009; Gantois et al., 2020; Gonzalez et al., 2024; Kürkcü Akgönül and Özen, 2025), as mental fatigue can impair both endurance and cognitive performance. As fatigue levels increase, groups with a higher resistance to fatigue display significant advantages in endurance and cognitive performance. The BET group, by contrast, demonstrated such advantages: while mental fatigue was exacerbated in the fresh state compared to the control group, the BET group showed progressively more pronounced improvements as fatigue levels increased. This finding aligns with previous research (Dallaway et al., 2024), which has demonstrated that brain endurance training can significantly improve performance in tasks sensitive to mental fatigue, particularly those affected by cognitive load.

Based on these results, we recommend that future studies further explore the impact of BET on performance tasks vulnerable to mental fatigue, particularly in the selection of performance evaluation metrics. However, it should be noted that this study did not fully present the data on Stroop test performance in the fatigued state, largely due to limitations in the sample size. Researchers should therefore interpret the current findings with caution and aim to conduct further validation and screening with larger sample sizes to enhance the reliability and generalizability of the conclusions. Nevertheless, given the advantage of BET in enhancing mental fatigue resistance, we can reasonably expect that the BET group will show significant improvements in Stroop response times under fatigued conditions compared to the control group.

In the studies included in our review, some (Staiano et al., 2022; de Lima-Junior et al., 2023; Staiano et al., 2023; Dallaway et al., 2024) employed a two-way interaction analysis of group and time, focusing on the effect changes before and after intervention for each group. In contrast, other studies (Díaz-García et al., 2023; Dallaway et al., 2023; Díaz-García et al., 2024; Díaz-García et al., 2025; Staiano et al., 2025) took a more refined analytical approach by incorporating a three-way interaction effect of group, time, and state (i.e., awake vs. fatigued) to investigate the impact on performance fatigability. These cognitive tasks primarily involve the Stroop task (Díaz-García et al., 2023; Díaz-García et al., 2024; Díaz-García et al., 2025; Staiano et al., 2025) or N-back tasks (Dallaway et al., 2023). These studies aimed to induce cognitive resource depletion in participants (Goodman et al., 2025) in order to examine differences in fatigue resistance across groups. This analytical framework is more comprehensive and rigorous. Limiting the analysis to the two-way interaction of group and time without considering the potential moderating effect of fatigue intervention on endurance and cognitive performance may lead to limited interpretation of the results. Thus, we recommend that future research prioritize three-way interaction analyses that include the state factor to more systematically reveal the mechanisms and applied value of Brain Endurance Training (BET). Additionally, the inclusion of state factors in the analysis provides a more nuanced view of BET’s performance-enhancing effects under complex conditions (André et al., 2025). Given practical application needs, future studies should focus on performance assessments under various fatigue states, particularly in conditions of physical fatigue or a combination of physical and mental fatigue.

It is important to note that the operational definitions of mental fatigue vary significantly across the included studies—ranging from subjective ratings to reaction-time decrements. This heterogeneity may explain the inconsistent findings regarding subjective outcomes. By distinguishing between subjective sensations and objective performance fatigability, our review highlights that BET is a potential strategy for enhancing ‘fatigue resilience’ rather than a tool for immediate subjective recovery. Future research should prioritize this three-way classification to systematically reveal the mechanisms of BET under complex fatigue states.

Currently, research on the subjective impact of BET on mental fatigue remains inconsistent (André et al., 2025). Our study provides new evidence on this issue. Our results indicate that, compared to the control group, BET did not reduce fatigue in the fresh state and may have even slightly increased it, which could be attributed to the subjective fatigue accumulated during the cognitive tasks involved in the training. However, in the fatigued state, the BET group was better able to maintain or reduce fatigue. In fact, under cognitive load, both the BET and control groups exhibited an increase in mental fatigue, but the increase in fatigue was significantly lower in the BET group than in the control group. We hypothesize that a possible mechanism of BET is supporting the brain’s ability to resist mental fatigue under high cognitive load. This suggests that the main benefit of BET is observed when the brain approaches its fatigue threshold, where it enhances the brain’s motor control or fatigue tolerance capacity. Specifically, this enhancement may be linked to improved neural efficiency under stress. As demonstrated by VanHaitsma et al (VanHaitsma et al., 2023), psychological or brain endurance training can lead to reduced electromyography (EMG) activity during time-to-exhaustion trials. This reduction likely reflects an optimization of motor control, where the central nervous system maintains the required physical output with lower neural drive to the working muscles. Consequently, during the final stages of long-duration competitions, traditional physical training may be sufficient to maintain performance in non-fatigued states, while BET provides a valuable strategy for gaining a competitive advantage by delaying central fatigue and preserving motor efficiency under extreme conditions.

Our meta-analysis results demonstrate that BET significantly improves endurance performance in both alert (SMD = 0.21, 95% CI 0.08–0.34) and fatigued states (SMD = 0.87, 95% CI 0.61–1.13) compared to control conditions. Interestingly, most included studies reported that the BET and control groups exhibited consistent external loads (Staiano et al., 2022; Díaz-García et al., 2023; Dallaway et al., 2023; Staiano et al., 2023; Díaz-García et al., 2024; Dallaway et al., 2024; Staiano et al., 2025)., suggesting that the observed performance gains might stem from adaptations within the central nervous system rather than peripheral changes alone. From a mechanistic perspective, although our meta-analysis focused on behavioral outcomes, existing theoretical models offer a plausible framework for these findings. It has been hypothesized that BET-induced cognitive load acts as a chronic stressor that modulates the brain’s perception of effort (Kolb and Whishaw, 1998; Marcora et al., 2015; Roelands and Bogataj, 2024). The anterior cingulate cortex (ACC) is known to play a pivotal role in effort-based decision-making and the integration of perceived exertion (Walton et al., 2002). While our study did not quantitatively synthesize neurophysiological data, external neuroimaging evidence suggests that BET may lead to functional adaptations in the ACC (Dallaway et al., 2023), potentially optimizing how effort is processed during prolonged physical tasks. Furthermore, supplementary cognitive training has been associated with increased activation and oxygenation of the prefrontal cortex (PFC) (Roelands and Bogataj, 2024). It is possible that repeated activation of these regions during BET could enhance neural efficiency or functional capacity, thereby delaying the onset of mental fatigue (Walton et al., 2002; Roelands and Bogataj, 2024). However, since the current meta-analysis did not include neurophysiological endpoints or correlation analyses between neural and behavioral data, these mechanistic interpretations remain speculative.

Although the subgroup analysis did not reveal significant differences between groups, the trends in significance levels and effect sizes suggest that the impact of BET on endurance performance may vary based on specific characteristics. Andre et al (André et al., 2025). reported that simultaneous tasks were more effective than sequential tasks. However, our findings suggest that this might not be the case. Regarding the timing of cognitive training, we found that cognitive training conducted before physical training achieved statistical significance. While we speculate that the results may be influenced by factors such as the dosage of cognitive tasks, the type of physical tasks, or sample characteristics, the underlying mechanisms remain unclear and warrant further investigation in future studies.

Regarding endurance testing measures, no significant moderating effects of BET were found; however, dynamic endurance tests appeared to better reflect the effects of BET. This finding is consistent with the study by Dallaway et al (Dallaway et al., 2024), who noted that BET primarily improves dynamic physical performance with limited effects on static tasks. This may be due to the greater influence of peripheral fatigue on static tasks. Our study further corroborated this result by including multiple studies in the analysis.

In terms of training levels, both trained and untrained populations showed improvements in endurance performance after training; however, statistically significant changes were only observed in the untrained group. This may be because trained athletes have already reached a high level of performance through regular training, and BET serves more as an optimization tool rather than a breakthrough intervention. In contrast, the untrained population, having lower baseline endurance, may benefit more from BET.

Regarding training-related variables, a longer intervention duration and lower training frequency were found to be more beneficial for enhancing endurance performance. A longer training period provides individuals with sufficient time to stimulate and influence the central nervous system, promoting combined adaptation of the central and peripheral systems. Additionally, lower training frequency helps prevent cognitive overload, allowing individuals to better maintain and recover, thus optimizing the training effect more effectively. Furthermore, this study found no significant moderation effect of gender on the outcomes of BET, indicating its potential applicability across different populations.

We also conducted a meta-regression analysis, which revealed no significant dose-response relationship between any variables and BET effects (*p >* 0.05). This may be due to the limited number of studies included and the considerable methodological differences across studies, which constrained the possibility for moderation analysis. Therefore, future research will need larger sample sizes to explore optimal thresholds and ranges through large-scale regression analyses, which will further facilitate practical applications and translations.

### Limitations of existing studies and this review

There are several significant limitations in the existing research on BET. First, studies on the effects of different training frequencies, durations, and repetitions on BET outcomes are still in the preliminary stages. The scarcity of existing research has led to low statistical power in the current meta-analysis, subgroup analyses, and meta-regression analyses, which may result in imprecise aggregated findings. Further dose-response studies are necessary, as these would provide important practical guidance. Additionally, some studies failed to standardize the reporting of outcome data (e.g., means and standard deviations), which made data extraction difficult and could introduce estimation biases in future meta-analyses, increasing the risk of bias. At the same time, sensitivity analyses showed that excluding certain studies led to noticeable changes in the effect estimates, and in some cases shifted results from significant to non-significant. This suggests that the findings may be influenced by a small number of studies. Therefore, the results should be interpreted with caution. Finally, due to small sample sizes, Egger’s test may produce false-positive bias results, and thus, these results should be interpreted with caution (Afonso et al., 2024).

The review also has several limitations: (1) The literature search was restricted to English-language studies. Due to a lack of corresponding data, some studies were excluded, potentially introducing publication bias, selection bias, and language bias; (2) There were variations in the test methods used to measure different skill levels, which increased the difficulty of making direct comparisons between studies and limited the generalizability of the conclusions. (3) As BET is an emerging field, its operational definition has remained somewhat ambiguous in prior original research. This lack of conceptual clarity may hinder the clear distinction between BET and other paradigms, such as dual-task training or general cognitive interventions. Such ambiguity poses challenges to a precise understanding of BET’s unique mechanisms and suggests that future research should establish more rigorous criteria to differentiate BET from related cognitive-physical training models.

## Conclusion

Brain Endurance Training (BET) may represent a promising approach for mitigating mental fatigue and enhancing both endurance and cognitive performance. Notably, BET appeared to show relatively more consistent effects under experimentally induced fatigue conditions, suggesting that its effects may be more evident in such contexts. Although subgroup analyses did not reveal statistically significant differences across moderating variables, observed trends in effect sizes suggest that BET may have a potential influence on individual endurance performance. Based on these exploratory findings, BET may be considered as a potential intervention to improve dynamic endurance performance. Interventions lasting longer than eight weeks and involving no more than four sessions per week appeared to be associated with relatively greater effects. In addition, cognitive training performed prior to physical training may be beneficial for enhancing overall training outcomes. However, these observations should be interpreted with caution due to the lack of statistically significant subgroup differences. Future research is warranted to further examine the effects of BET under different conditions, assess its resistance to mental fatigue across various states, and incorporate neuroimaging techniques to better elucidate the underlying mechanisms.

## Data Availability

The original contributions presented in the study are included in the article/**Supplementary Material**. Further inquiries can be directed to the corresponding author/s.
