# Supplementary material for: The effects of brain endurance training on mental fatigue, endurance, and cognitive function: a systematic review and meta-analysis
Source: Front Physiol. 2026 Jun 3;17:1746298. doi: 10.3389/fphys.2026.1746298 (PMC13272421; doi:10.3389/fphys.2026.1746298)
Supplement: Supplementary file 1 [file Table1.docx]

**SUPPLEMENTAL MATERIAL**

| **The Effects of Brain Endurance Training on Mental Fatigue, Endurance, and Cognitive Function: A Systematic Review and Meta-Analysis** |
| --- |

[Table S1. Characteristics of brain endurance training（BET）interventions included in the meta-analysis. 2](#_Toc18255)

[Table S2. Search strategy detailed for each database. 9](#_Toc9910)

[Table S3. PEDro Results 11](#_Toc12828)

[Table S4. Results of meta-regression in search of possible moderators. 16](#_Toc7403)

[Covariate: total duration of each training session; 16](#_Toc22460)

[Covariate: frequency of training per week 17](#_Toc17037)

[Covariate: total training duration per week 17](#_Toc441)

[Covariate: number of training weeks 18](#_Toc4360)

[Covariate: total training duration 19](#_Toc22931)

[Covariate: duration of each physical training session 20](#_Toc17849)

[Covariate: duration of each cognitive training session 21](#_Toc29795)

[Covariate: total number of training sessions 22](#_Toc447)

[Covariate: age 23](#_Toc30095)

Table S1. Characteristics of brain endurance training（BET）interventions included in the meta-analysis.

|  |  |  | **n** | |  | **Age** |  | **Training intervention** | | | | | **Outcomes** | | | | |
| --- | --- | --- | --- | --- | --- | --- | --- | --- | --- | --- | --- | --- | --- | --- | --- | --- | --- |
|  |  |  |  |  |  |  |  |  |  |  | |  |  |  | |  |  |
| **Study，year** | **Group** | **Sport** | **Group f（%）** | **Subjects** |  | **Mean（±SD)** |  | **Fre(times·wk^-1^）** | **Duration** | **BET** | | **CT** | **Endurance** | **Stroop-RT** | | **Pvt-Rt** | **Vas-mf** |
| Staiano et al. 2022 | a | Soccer | 0 | T=13  C=12 |  | 22.4±4.3 |  | 4-5 | 4 | Completed 40 physical training sessions within 4 weeks (1-2 sessions per day, 5 days a week), including regular football training (e.g., technical, tactical, and fitness training). | Post，Go/No-Go、AX-CPT | Identical to the experimental group. |  | 30min Stroop RT | | 10minPVT-B |  |
| Staiano et al. 2023 （a） | a | Road cycling | 0 | T=13  C=13 |  | 29±5 |  | 5 | 6 | 4 cycling sessions per week + 1 strength and conditioning session. | Post，Flanker Task、Go/No-Go、AX-CPT | Identical to the experimental group. | TTE | 30min Stroop RT |  | |  |
| Staiano et al. 2023（b） | a | Road cycling | 0 | T=13  C=11 |  | 25±4 |  | 5 | 6 | 4 cycling sessions per week + 1 strength and conditioning session. | Post | Identical to the experimental group. | TT | 30min Stroop RT |  | |  |
| Staiano et al. 2025 | a | Soccer | 0 | T=15  C=16 |  | 26.32±8.41 |  | 3 | 6 | Warm-up (4 minutes), passing drills (10 minutes), large-sided game (12 minutes), tactical training (12 minutes), simulated match (12 minutes), etc | Mixed，Flanker Task、Go/No-Go、AX-CPT | Identical to the experimental group. |  |  | 3minPVT-B | | 10cm VAS-MF |
| Díaz et al. 2024 | a | resistance-trained athletes | T=43.5%  C=44.4% | T=46  C=45 |  | 29.42±10 |  | 5 | 6 | Each training session includes 4 sets of bench presses (40% of 6RM intensity to failure) and 4 sets of squat jumps (to failure); warm-up consists of 10 minutes of cycling (60% of maximum heart rate), and cool-down includes 15 minutes of cycling (85% of maximum heart rate). | Mixed，Stroop | Identical to the experimental group. | CHEST PRESS REPETITIONS  、  SJ REPETITIONS  PVT RT |  | 3minPVT-B | | 10cm VAS-MF |
| Díaz et al. 2023 | a | Tennis | 0 | T=30  C=31 |  | 28.71 ± 7.68 |  | 3 | 6 | 10-minute warm-up, 15 minutes of technical training (coach-fed ball exercises for intercepting/striking), 15 minutes of tactical training (specific constraint drills), and 20 minutes of simulated match. | Mixed，incongruent Stroop task | Identical to the experimental group. |  |  | 3minPVT-B | | 10cm VAS-MF |
| Díaz et al. 2025 | b |  | 100% | T=8  C1=8  C2=8 |  | 71.42±4.02 |  | 3 | 8 | 20 minutes of resistance training (1 minute of squats + 1.5 minutes rest + 1 minute of bicep curls + 1.5 minutes rest, repeated for 4 sets) and 25 minutes of outdoor walking. | Pre，incongruent Stroop task | **C1:** "20 minutes of resistance training (1 minute of squats + 1.5 minutes rest + 1 minute of bicep curls + 1.5 minutes rest, repeated for 4 sets) and 25 minutes of outdoor walking."  **C2:** "None." | 6-minute walking distance、30-second chair stand repetitions、and 30-second arm curl repetitions | 45 s Stroop RT | 3minPVT-B | | 10cm VAS-MF |
| de Lima et al. 2023 | a | runners | 0 | T=15  C1=15  C2=15 |  | 23.4±2.9 |  | 3 | 12 | Treadmill running at 60% Δ maximum aerobic speed (Vmax), with duration starting at 20 minutes (weeks 1-2) and gradually increasing to 40 minutes (weeks 11-12), with a 5-minute increase every 2 weeks. | Current，Stroop | **C1:** "Completed only the same Stroop task as the CET group, with identical duration and intensity progression, no running training."  **C2:** "Completed only the same running training as the CET group (identical intensity, duration, and progression), no cognitive tasks." | Tte | 300 times Stroop RT |  | |  |
| Dallaway et al. 2024（a） | b |  | T=42.9%  C=40% | T=14  C=15 |  | 23±5 |  | 3 | 4 | 2 sets of plank, squats, push-ups, and wall sit. | Mixed，Stroop、2-back、AX-CPT | Identical to the experimental group. | PRESS UP  、WALL SIT  、PLANK |  |  | | 10cm VAS-MF |
| Dallaway et al. 2024（b） | b |  | T=66.7%  C=64.3% | T=14  C=15 |  | 21±2 |  | 3 | 4 | 1 set of burpees, plank, squat jumps, leg raises, and push-ups. | Mixed，2-back、Time-Load Dual-Back、Switched Stop Visual、Multi-Source Interference Task | Identical to the experimental group. | BURPEES  JUMP SQUATS  LEG RAISES  PRESS UPS  PLANK |  |  | | 10cm VAS-MF |
| Dallaway et al. 2023 | b |  | T=54.2%  C=54.2% | T=12  C=12 |  | 20±2 |  | 4 | 5 | Complete rhythmic grip strength task (30% of maximum voluntary contraction intensity, gripping to the beat of a metronome at 1 grip per second, reaching a preset cumulative force target, with weekly target increases of 1000  points). | Pre，2-back、Stroop | Identical to the experimental group. | AVERAGE FORCE |  |  | | 10cm VAS-MF |

BET: Brain Endurance Training；CT: Control Group；VAS-mf: Visual Analogue Scale for Mental Fatigue；Stroop-RT: Stroop Reaction Time Task；PVT-RT: Psychomotor Vigilance Task Reaction Time；TTE: Time to Exhaustion；TT: Time Trial；Vmax: Maximum Aerobic Speed；6RM: Six Repetition Maximum；PVT-B: Psychomotor Vigilance Test - B；CET: Cognitive Training Group；Pre: Cognitive task performed before training；Post: Cognitive task performed after training；Current: Cognitive task performed simultaneously with training；Mixed: Cognitive task performed intermittently during training；C1, C2: Group 1, Group 2；T: Experimental Group (with intervention)；C: Control Group (without intervention)；Group a: Athletes with more than 2 years of training experience；Group b: Athletes with less than 2 years of training experience；Group f(%): Percentage of female participants in each group；Subjects: Number of participants；Mean (±SD): Mean value ± Standard Deviation；Fre (times·wk-1): Frequency of training per week；Duration: Duration of training (in weeks)；Training Intervention: Type of training intervention (e.g., BET, resistance training)；Outcomes: Measured outcomes (e.g., endurance, Stroop-RT, PVT-RT, VAS-mf)；

Table S2. Search strategy detailed for each database.

| **Database** | **Full search strategy** |
| --- | --- |
| **PubMed** | (((((((((("Endurance Performance"[Title/Abstract]) OR ("Physical Endurance"[Title/Abstract])) OR ("Exercise Performance"[Title/Abstract])) OR ("Athletic Performance"[Title/Abstract])) OR ("Performance"[Title/Abstract])) OR ("Sport Performance"[Title/Abstract])) OR ("Aerobic Capacity"[Title/Abstract])) OR ("Physical Capacity"[Title/Abstract])) OR ("Mental Fatigue"[Title/Abstract])) OR ("Cognitive Fatigue"[Title/Abstract])) AND ((((("Brain training"[Title/Abstract]) OR ("BET"[Title/Abstract])) OR ("brain endurance training"[Title/Abstract])) OR ("Cognitive Training"[Title/Abstract])) OR ("Mental Endurance"[Title/Abstract])) |
| **Web of Sciences (all databases)** | (((((((((TS=("Endurance Performance")) OR TS=("Physical Endurance")) OR TS=("Exercise Performance")) OR TS=("Athletic Performance")) OR TS=("Performance")) OR TS=("Sport Performance")) OR TS=("Aerobic Capacity")) OR TS=("Physical Capacity")) OR TS=("Mental Fatigue")) OR TS=("Cognitive Fatigue") AND ((((TS=("Brain training")) OR TS=("BET")) OR TS=("brain endurance training")) OR TS=("Cognitive Training")) OR TS=("Mental Endurance") |
| **Scopus** | ( ( TITLE-ABS-KEY ( "Brain training" ) OR TITLE-ABS-KEY ( "BET" ) OR TITLE-ABS-KEY ( "brain endurance training" ) OR TITLE-ABS-KEY ( "Cognitive Training" ) OR TITLE-ABS-KEY ( "Mental Endurance" ) ) ) AND ( ( TITLE-ABS-KEY ( "Endurance Performance" ) OR TITLE-ABS-KEY ( "Physical Endurance" ) OR TITLE-ABS-KEY ( "Exercise Performance" ) OR TITLE-ABS-KEY ( "Athletic Performance" ) OR TITLE-ABS-KEY ( "Performance" ) OR TITLE-ABS-KEY ( "Sport Performance" ) OR TITLE-ABS-KEY ( "Aerobic Capacity" ) OR TITLE-ABS-KEY ( "Physical Capacity" ) OR TITLE-ABS-KEY ( "Mental Fatigue" ) OR TITLE-ABS-KEY ( "Cognitive Fatigue" ) ) ) |

| Table S3. PEDro Results | | |  |  |  |  |  |  |  |  |  |  |  |  |  |  |
| --- | --- | --- | --- | --- | --- | --- | --- | --- | --- | --- | --- | --- | --- | --- | --- | --- |
| **Standard** | **Dallaway 2023** | **Dallaway 2024** | **de Lima-Junior 2023** | **Díaz-García 2023** | **Díaz-García 2024** | **Díaz-García 2025** | **Staiano 2023** | **Staiano 2022** | **Staiano 2025** |  |  |  |  |  |  |  |
| 1. Eligibility criteria clearly defined | y | y | y | y | y | y | y | y | y |  |  |  |  |  |  |  |
| 2. Participants were randomly assigned to groups | 1 | 1 | 1 | 1 | 1 | 1 | 1 | 1 | 1 |  |  |  |  |  |  |  |
| 3. Allocation process was concealed | 0 (only mentions random assignment, does not specify methods like sealed envelopes or central randomization system) | 0 (does not describe allocation concealment measures) | 0 (only mentions stratified randomization using random number table, does not explain concealment method) | 0 (does not mention allocation concealment) | 0 (does not describe allocation concealment) | 0 (does not explain allocation concealment measures) | 0 (does not mention allocation concealment) | 0 (only random assignment, no concealment details) | 0 (does not mention allocation concealment) |  |  |  |  |  |  |  |
| 4. Groups were similar at baseline for key prognostic indicators | 1 (no baseline differences in key indicators such as age, gender, exercise habits, baseline fitness) | 1 (no significant baseline differences in demographics and fitness) | 1 (no baseline differences in age, weight, height, 10 km time, VO₂max) | 1 (no significant baseline differences in age, height, weight, training experience) | 1 (baseline key indicators like exercise performance and demographics balanced) | 1 (no baseline differences in cognitive and physical performance, demographics) | 1 (no significant baseline differences in VO₂peak, PPO, fitness) | 1 (no baseline differences in exercise ability and cognitive levels) | 0 (did not report baseline comparison of key prognostic indicators) |  |  |  |  |  |  |  |
| 5. All participants were blinded to treatment assignment | 0 (participants knew their training group; BET group had cognitive tasks, control group rested, no blinding) | 0 (participants knew their training tasks, no blinding) | 0 (participants knew their training type, no blinding) | 0 (participants knew their training group, BET group had cognitive tasks, control group rested) | 0 (participants knew training group and tasks, no blinding) | 0 (participants knew their group assignment, no blinding) | 0 (participants knew their training group, no blinding) | 0 (participants knew training content, no blinding) | 0 (no mention of blinding, training differences easy to identify, no blinding) |  |  |  |  |  |  |  |
| 6. All therapists were blinded to treatment assignment | 0 (therapists knew the training difference between BET and control groups) | 0 (therapists knew the training differences and guided accordingly, no blinding) | 0 (therapists knew the training protocols, no blinding) | 0 (therapists knew BET group tasks and control group tasks, no blinding) | 0 (therapists knew training differences and protocols, no blinding) | 0 (therapists knew the group assignments and protocols, no blinding) | 0 (therapists guided according to group tasks, knew the assignments) | 0 (therapists knew the differences in tasks between groups, no blinding) | 0 (no mention of blinding for therapists, unable to blind given training task differences) |  |  |  |  |  |  |  |
| 7. All assessors were blinded to treatment assignment | 1 (some assessments like grip strength, NIRS were conducted by blinded assessors, mentioned blinding for assessors) | 1 (motor performance assessments like repetitions, duration were done by blinded assessors) | 0 (no mention of blinding for assessors) | 1 (assessments like hitting speed, accuracy were done by blinded assessors) | 1 (physical performance tests like repetitions were performed by blinded assessors) | 1 (cognitive and physical assessments were conducted by blinded assessors) | 1 (motor performance and cognitive tests were completed by blinded assessors) | 1 (motor ability and cognitive tests were conducted by blinded assessors) | 0 (no mention of blinding for assessors) |  |  |  |  |  |  |  |
| 8. At least one key outcome measure had data from ≥ 85% of the initially assigned participants | 1 (initial sample size 24, no dropouts, data completeness 100%) | 1 (initial sample size 29, no dropouts, data completeness 100%) | 1 (initial sample size 45, no dropouts, data completeness ≥85%) | 1 (initial sample size 61, no dropouts, data completeness 100%) | 1 (initial sample size 91, no dropouts, data completeness 100%) | 1 (initial sample size 24, no dropouts, data completeness 100%) | 1 (Study 1 initial 28, 2 dropouts, completeness 92.9%; Study 2 initial 25, 1 dropout, completeness 96%) | 1 (initial 25, 3 dropouts, completeness 88%) | 0 (no report of initial sample size or dropout rate, unable to assess) |  |  |  |  |  |  |  |
| 9. All participants with outcome data were analyzed according to their assigned treatment or by intention-to-treat (ITT) analysis | 1 (no dropouts, all participants completed treatment as assigned, data analyzed according to actual group) | 1 (no dropouts, all participants completed training as assigned, data analyzed according to group) | 1 (no dropout reported, participants completed treatment as assigned, data analyzed) | 1 (no dropouts, all participants completed training as assigned, data analyzed) | 1 (no dropouts, participants completed treatment as assigned, data analyzed) | 1 (no dropouts, participants completed training as assigned, data analyzed) | 1 (dropouts had no data, remaining participants analyzed according to assignment) | 1 (dropouts had no data, remaining participants analyzed according to group) | 0 (no report on participant adherence or analysis methods) |  |  |  |  |  |  |  |
| 10. At least one key outcome measure had reported group statistical comparisons | 1 (ANOVA/t-test results reported for endurance performance, prefrontal oxygenation) | 1 (ANOVA results for motor performance, cognitive ability) | 1 (ANOVA results for VO₂max, exhaustion time, inhibition control) | 1 (ANOVA results for hitting speed, accuracy) | 1 (ANOVA results for resistance exercise repetitions, mental fatigue) | 1 (ANOVA results for cognitive and physical performance) | 1 (ANOVA results for endurance performance, cognitive response) | 1 (ANOVA results for motor ability, cognitive performance) | 0 (no reported statistical comparisons) |  |  |  |  |  |  |  |
| 11. Study provided at least one key outcome measure’s point estimate and variability measure | 1 (provided endurance performance improvement percentage, prefrontal oxygenation mean ± SD) | 1 (provided motor repetitions, cognitive response time mean ± SD) | 1 (provided VO₂max, exhaustion time mean ± SD, percentage change) | 1 (provided hitting speed, accuracy mean ± SE) | 1 (provided motor repetitions, reaction time mean ± SE) | 1 (provided cognitive scores, physical performance mean ± SE) | 1 (provided exhaustion time, reaction time mean ± SD) | 1 (provided motor speed, cognitive test scores mean ± SD) | 0 (no point estimate or variability provided for key outcomes) |  |  |  |  |  |  |  |

| Table S4. Results of meta-regression in search of possible moderators. | |
| --- | --- |
|  |  |
| **Covariate: total duration of each training session;** |  |
| linear |  |
| Model Results: |  |
|  |  |
| **estimate se tval df pval ci.lb ci.ub** |  |
| **intrcpt 0.0163 0.1705 0.0955 27 0.9246 -0.3335 0.3661** |  |
| **a 0.0390 0.0292 1.3346 27 0.1931 -0.0210 0.0990** |  |
|  |  |
|  |  |
| Polynomial Regression |  |
| Model Results: |  |
|  |  |
| **estimate se tval df pval ci.lb ci.ub** |  |
| **intrcpt -0.0766 0.4236 -0.1809 26 0.8579 -0.9473 0.7941** |  |
| **a 0.0717 0.1374 0.5219 26 0.6062 -0.2107 0.3541** |  |
| **I(a^2) -0.0024 0.0096 -0.2448 26 0.8085 -0.0221 0.0174** |  |
|  |  |
|  |  |
| Piecewise Linear Regression |  |
| Model Results: |  |
|  |  |
| **estimate se tval df pval ci.lb ci.ub** |  |
| **intrcpt 0.0369 0.4938 0.0747 25 0.9411 -0.9801 1.0539** |  |
| **rcs(a, 3)a 0.0342 0.1264 0.2705 25 0.7890 -0.2261 0.2945** |  |
| **rcs(a, 3)a' 0.0430 0.5429 0.0792 25 0.9375 -1.0752 1.1612** |  |
| **rcs(a, 3)a'' -0.1024 1.1802 -0.0868 25 0.9315 -2.5330 2.3282** |  |
|  |  |
|  |  |
| **Covariate: frequency of training per week** |  |
| linear |  |
| Model Results: |  |
|  |  |
| **estimate se tval df pval ci.lb ci.ub** |  |
| **intrcpt 0.6434 0.2731 2.3561 27 0.0260 0.0831 1.2036 *** |  |
| **b -0.1095 0.0673 -1.6262 27 0.1155 -0.2477 0.0287** |  |
|  |  |
|  |  |
|  |  |
|  |  |
|  |  |
| **Covariate: total training duration per week** |  |
| linear |  |
| Model Results: |  |
|  |  |
| **estimate se tval df pval ci.lb ci.ub** |  |
| **intrcpt 0.3771 0.1783 2.1150 27 0.0438 0.0113 0.7429 *** |  |
| **c -0.0037 0.0038 -0.9692 27 0.3411 -0.0115 0.0041** |  |
|  |  |
|  |  |
| Polynomial Regression |  |
|  |  |
| Model Results: |  |
|  |  |
| **estimate se tval df pval ci.lb ci.ub** |  |
| **intrcpt -0.1214 0.3906 -0.3107 26 0.7585 -0.9242 0.6815** |  |
| **c 0.0311 0.0245 1.2666 26 0.2165 -0.0193 0.0814** |  |
| **I(c^2) -0.0005 0.0003 -1.4418 26 0.1613 -0.0011 0.0002** |  |
|  |  |
|  |  |
| **Covariate: number of training weeks** |  |
| linear |  |
| Model Results: |  |
|  |  |
| **estimate se tval df pval ci.lb ci.ub** |  |
| **intrcpt 0.3815 0.1350 2.8246 27 0.0088 0.1044 0.6586 **** |  |
| **d -0.0006 0.0004 -1.4353 27 0.1627 -0.0015 0.0003** |  |
|  |  |
| Polynomial Regression |  |
|  |  |
| Model Results: |  |
|  |  |
| **estimate se tval df pval ci.lb ci.ub** |  |
| **intrcpt 0.2141 0.3195 0.6701 26 0.5087 -0.4427 0.8708** |  |
| **d 0.0011 0.0030 0.3715 26 0.7133 -0.0051 0.0074** |  |
| **I(d^2) -0.0000 0.0000 -0.5780 26 0.5682 -0.0000 0.0000** |  |
|  |  |
|  |  |
| Piecewise Linear Regression |  |
|  |  |
| Model Results: |  |
|  |  |
| **estimate se tval df pval ci.lb ci.ub** |  |
| **intrcpt 0.1948 0.2747 0.7093 26 0.4844 -0.3698 0.7594** |  |
| **rcs(d, 3)d 0.0009 0.0020 0.4515 26 0.6554 -0.0032 0.0049** |  |
| **rcs(d, 3)d' -0.0027 0.0034 -0.7803 26 0.4423 -0.0096 0.0043** |  |
|  |  |
|  |  |
|  |  |
| **Covariate: total training duration** |  |
| linear |  |
|  |  |
| Model Results: |  |
|  |  |
| **estimate se tval df pval ci.lb ci.ub** |  |
| **intrcpt 0.2275 0.1378 1.6510 27 0.1103 -0.0552 0.5102** |  |
| **e 0.0000 0.0001 0.0874 27 0.9310 -0.0002 0.0002** |  |
|  |  |
| Piecewise Linear Regression |  |
|  |  |
|  |  |
| Variance Components: |  |
|  |  |
| **estim sqrt nlvls fixed factor** |  |
| **sigma^2.1 0.0131 0.1142 7 no studyid** |  |
| **sigma^2.2 0.0000 0.0000 29 no esid** |  |
|  |  |
|  |  |
| Model Results: |  |
|  |  |
| **estimate se tval df pval ci.lb ci.ub** |  |
| **intrcpt 0.2415 0.2399 1.0067 26 0.3233 -0.2516 0.7347** |  |
| **rcs(e, 3)e -0.0000 0.0003 -0.0432 26 0.9659 -0.0006 0.0006** |  |
| **rcs(e, 3)e' 0.0000 0.0004 0.0905 26 0.9286 -0.0009 0.0010** |  |
|  |  |
|  |  |
|  |  |
| Polynomial Regression |  |
|  |  |
| Model Results: |  |
|  |  |
| **estimate se tval df pval ci.lb ci.ub** |  |
| **intrcpt 0.2108 0.2512 0.8391 26 0.4091 -0.3055 0.7271** |  |
| **e 0.0000 0.0004 0.0962 26 0.9241 -0.0008 0.0008** |  |
| **I(e^2) -0.0000 0.0000 -0.0583 26 0.9540 -0.0000 0.0000** |  |
|  |  |
| **Covariate: duration of each physical training session** |  |
| linear |  |
|  |  |
| Model Results: |  |
|  |  |
| **estimate se tval df pval ci.lb ci.ub** |  |
| **intrcpt 0.3689 0.2648 1.3929 27 0.1750 -0.1745 0.9122** |  |
| **f -0.0061 0.0110 -0.5564 27 0.5825 -0.0288 0.0165** |  |
|  |  |
|  |  |
| Piecewise Linear Regression |  |
|  |  |
| Model Results: |  |
|  |  |
| **estimate se tval df pval ci.lb ci.ub** |  |
| **intrcpt 0.6031 0.3681 1.6385 26 0.1134 -0.1535 1.3597** |  |
| **rcs(f, 3)f -0.0185 0.0174 -1.0686 26 0.2951 -0.0542 0.0171** |  |
| **rcs(f, 3)f' 0.0124 0.0163 0.7611 26 0.4534 -0.0211 0.0459** |  |
|  |  |
| Polynomial Regression |  |
|  |  |
| Model Results: |  |
|  |  |
| **estimate se tval df pval ci.lb ci.ub** |  |
| **intrcpt 0.5734 0.6747 0.8498 26 0.4032 -0.8135 1.9602** |  |
| **f -0.0232 0.0530 -0.4372 26 0.6656 -0.1321 0.0857** |  |
| **I(f^2) 0.0003 0.0010 0.3262 26 0.7469 -0.0017 0.0024** |  |
|  |  |
| **Covariate: duration of each cognitive training session** |  |
|  |  |
| linear |  |
|  |  |
| Model Results: |  |
|  |  |
| **estimate se tval df pval ci.lb ci.ub** |  |
| **intrcpt 0.4031 0.1875 2.1496 27 0.0407 0.0183 0.7880 *** |  |
| **g -0.0029 0.0027 -1.0784 27 0.2904 -0.0085 0.0026** |  |
|  |  |
|  |  |
| Model Results: |  |
|  |  |
| **estimate se tval df pval ci.lb ci.ub** |  |
| **intrcpt 0.1276 0.3253 0.3922 26 0.6981 -0.5411 0.7963** |  |
| **rcs(g, 3)g 0.0048 0.0079 0.6053 26 0.5502 -0.0115 0.0211** |  |
| **rcs(g, 3)g' -0.0087 0.0083 -1.0404 26 0.3077 -0.0258 0.0084** |  |
|  |  |
|  |  |
|  |  |
| **Covariate: total number of training sessions** |  |
|  |  |
| linear |  |
|  |  |
| Model Results: |  |
|  |  |
| **estimate se tval df pval ci.lb ci.ub** |  |
| **intrcpt 0.1372 0.1860 0.7377 27 0.4671 -0.2444 0.5189** |  |
| **h 0.0050 0.0083 0.6021 27 0.5521 -0.0121 0.0222** |  |
|  |  |
|  |  |
| Piecewise Linear Regression |  |
|  |  |
| Model Results: |  |
|  |  |
| **estimate se tval df pval ci.lb ci.ub** |  |
| **intrcpt 0.0682 0.6191 0.1101 26 0.9131 -1.2043 1.3407** |  |
| **rcs(h, 3)h 0.0104 0.0456 0.2288 26 0.8208 -0.0833 0.1041** |  |
| **rcs(h, 3)h' -0.0154 0.1351 -0.1142 26 0.9100 -0.2932 0.2623** |  |
|  |  |
| Polynomial Regression |  |
|  |  |
| Model Results: |  |
|  |  |
| **estimate se tval df pval ci.lb ci.ub** |  |
| **intrcpt 0.1225 0.7071 0.1732 26 0.8638 -1.3310 1.5759** |  |
| **h 0.0067 0.0738 0.0908 26 0.9284 -0.1450 0.1584** |  |
| **I(h^2) -0.0000 0.0017 -0.0194 26 0.9847 -0.0034 0.0034** |  |
|  |  |
|  |  |
| **Covariate: age** |  |
|  |  |
| linear |  |
|  |  |
| Model Results: |  |
|  |  |
| **estimate se tval df pval ci.lb ci.ub** |  |
| **intrcpt 0.1890 0.1707 1.1071 27 0.2780 -0.1612 0.5392** |  |
| **i 0.0014 0.0043 0.3234 27 0.7489 -0.0074 0.0102** |  |
|  |  |
| Piecewise Linear Regression |  |
|  |  |
| Model Results: |  |
|  |  |
| **estimate se tval df pval ci.lb ci.ub** |  |
| **intrcpt 1.0244 0.5593 1.8317 26 0.0785 -0.1252 2.1740 .** |  |
| **rcs(i, 3)i -0.0326 0.0216 -1.5149 26 0.1419 -0.0769 0.0117** |  |
| **rcs(i, 3)i' 0.1059 0.0650 1.6284 26 0.1155 -0.0278 0.2395** |  |
|  |  |
| Polynomial Regression |  |
|  |  |
| Model Results: |  |
|  |  |
| **estimate se tval df pval ci.lb ci.ub** |  |
| **intrcpt 1.3978 0.7835 1.7842 26 0.0861 -0.2126 3.0082 .** |  |
| **i -0.0651 0.0414 -1.5743 26 0.1275 -0.1501 0.0199** |  |
| **I(i^2) 0.0007 0.0004 1.6277 26 0.1156 -0.0002 0.0016** |  |
